# A KMT2C loss-of-function mutation in a JAK2-negative polycythemia vera–like myeloproliferative neoplasm: a case report

**DOI:** 10.3389/fonc.2026.1825434

**Published:** 2026-04-28

**Authors:** Tala N. Musa, Jalal Al Khateeb, Yousef Sajdieh, Nasser M. Abualia, Akram Karama

**Affiliations:** Faculty of Medicine, Al-Quds University, East Jerusalem, Palestine

**Keywords:** clonal hematopoiesis, hydroxyurea, JAK2-negative, KMT2C, MLL3, myeloproliferative neoplasm, next-generation sequencing, polycythemia vera

## Abstract

**Background:**

Polycythemia vera (PV) is a clonal myeloproliferative neoplasm (MPN) typically defined by JAK2 mutations. JAK2-negative presentations pose significant diagnostic challenges and likely harbor a spectrum of molecular drivers that remain incompletely characterized.

**Case presentation:**

We report a case of JAK2-negative PV-like MPN in a 53-year-old male presenting with erythrocytosis, suppressed erythropoietin, panmyelotic bone marrow morphology, and splenic vein thrombosis. Standard JAK2 V617F and exon 12 analyses were negative. Next-generation sequencing (NGS) identified a KMT2C nonsense variant (c.2961C>G, p.Tyr987*) at a variant allele frequency (VAF) of 21%, providing molecular evidence of clonality. The clinical course was complicated by intermittent hydroxyurea non-adherence with marked hematocrit fluctuations; dose optimisation from 1 g to 2 g daily achieved stable hematological control.

**Conclusion:**

This case adds to the emerging molecular landscape of JAK2-negative MPNs by identifying KMT2C loss-of-function as a clonal marker in a patient with a JAK2-negative PV-like MPN phenotype. Whether KMT2C p.Tyr987* contributes causally to the erythroid-biased expansion or represents an accompanying clonal event warrants functional investigation. Comprehensive genomic profiling is essential when canonical driver mutations are absent, and strict adherence to cytoreductive targets remains critical to prevent thrombotic complications.

## Introduction

Polycythemia vera (PV) is a clonal myeloproliferative neoplasm (MPN) characterized by constitutive activation of the JAK-STAT signaling pathway, resulting in trilineage hyperplasia with predominant erythrocytosis. Acquired mutations in the JAK2 gene underlie approximately 95% of cases, most commonly the V617F point mutation, which serves as the cornerstone of molecular diagnosis (1). The 2022 World Health Organization (WHO) classification and the 2023 International Consensus Classification (ICC) both retain JAK2 mutational status as a major diagnostic criterion for PV, alongside hemoglobin/hematocrit thresholds and characteristic bone marrow morphology with trilineage proliferation and pleomorphic megakaryocytes (1–4).

The minority of patients who present with a clinical and hematologic phenotype fully consistent with PV yet lack canonical JAK2 mutations pose substantial diagnostic challenges. In such cases, an integrative approach incorporating bone marrow histology, suppressed serum erythropoietin (EPO), and systematic exclusion of secondary erythrocytosis is required. Importantly, the absence of JAK2 mutations necessitates explicit consideration of alternative diagnostic categories, including clonal erythrocytosis, atypical MPN, MPN-unclassifiable, and other non-canonical molecular entities, before a PV diagnosis is affirmed (1, 2).

We report a case in which a pathogenic KMT2C loss-of-function variant was identified as the sole detectable molecular marker of clonality in a patient with clinical and morphological features overlapping with PV but not meeting full molecular diagnostic criteria under the 2022 WHO framework, in the absence of JAK2, CALR, or MPL mutations. KMT2C encodes the histone H3K4 methyltransferase MLL3, a recognized tumor suppressor implicated in myeloid and solid malignancies (5, 6). To our knowledge, this represents a previously unreported association of a KMT2C loss-of-function mutation with a JAK2-negative PV-like MPN phenotype. This report contributes a novel molecular observation to the expanding landscape of JAK2-negative MPNs and illustrates the diagnostic utility of comprehensive genomic profiling in atypical presentations. The association observed is correlative; the mechanistic contribution of KMT2C p.Tyr987* to the erythroid phenotype remains to be established through functional studies.

## Case presentation

### Patient information

A 53-year-old male of Middle Eastern descent with a 15-pack-year smoking history and no prior hematologic diagnosis or family history of hematologic or thrombotic disorders presented in early 2018 with abdominal pain. Emergency department records from 2014 documented non-specific chest pain with entirely normal blood counts at that time.

### Primary concerns and clinical findings

**Figure 1:**
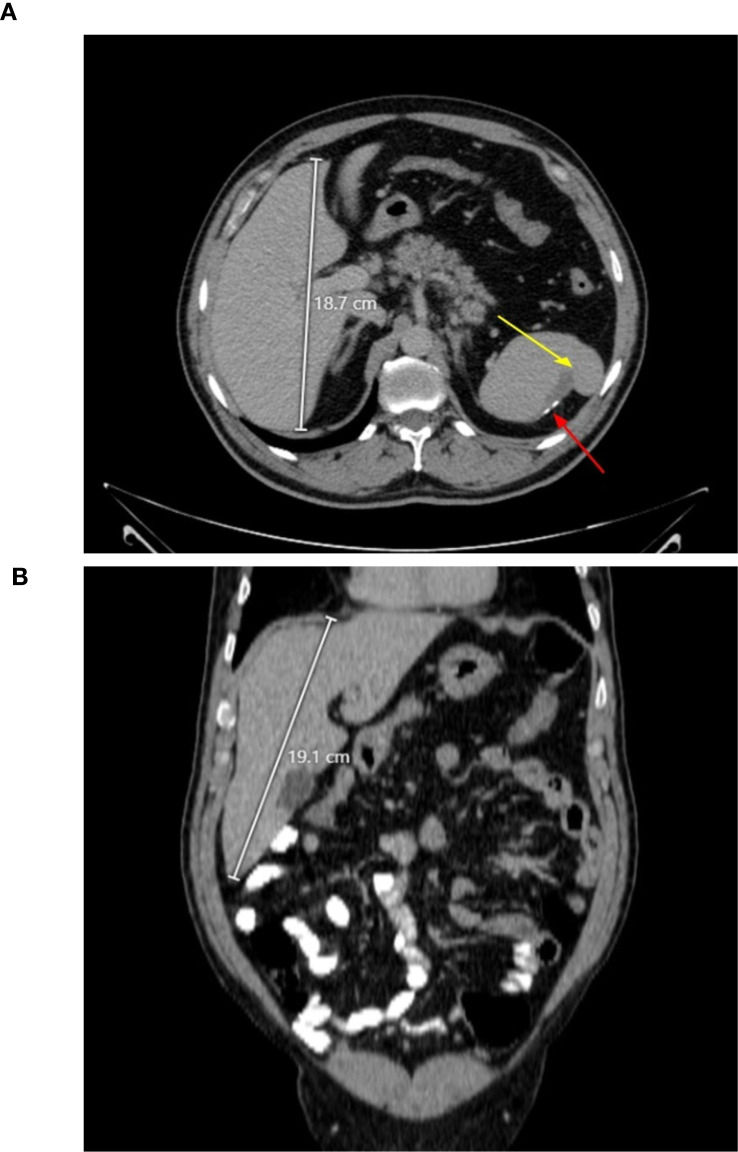
Axial **(A)** and coronal **(B)** abdominal CT scan demonstrating hepatomegaly. The spleen appears normal in size. **(A)** A hypodense splenic lesion (yellow arrow) indicates prior infarction; foci of calcification (red arrow) are consistent with post-infarct changes. **(B)** The liver measures 19.1 cm in maximal craniocaudal dimension (white line), consistent with significant hepatomegaly.

In January 2018, the patient was evaluated for lower abdominal pain, headache, and persistent low back pain radiating to the right thigh. Abdominal ultrasound incidentally identified a 1.7 cm splenic lesion, initially characterized as cystic but subsequently demonstrating features of chronic infarction, likely thrombotic in origin. Hepatic and renal morphology was otherwise unremarkable. Over subsequent years, he reported recurrent episodes of abdominal and flank pain with intermittent nausea and exertional dyspnea, with serial imaging revealing progressive hepatosplenic involvement. A CT scan obtained in July 2022 demonstrated significant hepatomegaly (craniocaudal dimension 19.1 cm) and a hypodense splenic lesion with internal calcifications consistent with prior infarction (**Figure 1**).

Initial laboratory evaluation in 2018 revealed persistent erythrocytosis with a hemoglobin concentration of 18 g/dL, accompanied by mild leukocytosis and normal platelet counts. On serial full blood counts, hemoglobin ranged from 13.7 to 19.4 g/dL and hematocrit rose to 60%; leukocyte counts were intermittently elevated while platelet counts remained within the reference range throughout.

### Relevant past interventions and outcomes

Prior to the index presentation, the patient had received no hematologic treatment. Following diagnosis, hydroxyurea 1 g daily and low-dose aspirin 100 mg daily were initiated. Adherence was intermittent: during periods of adherence, hematological parameters normalized (hemoglobin 13.7 g/dL, hematocrit 38%); lapses resulted in recurrent erythrocytosis (hemoglobin 19.4 g/dL, hematocrit 57%), necessitating urgent therapeutic phlebotomy. The hydroxyurea dose was subsequently adjusted to 2 g daily to maintain a target hematocrit below 45%. Repeated counseling for smoking cessation was provided throughout.

### Clinical timeline

**Table 1** summarises key clinical events and investigations from the patient’s episode of care.

**Table 1:** Patient clinical timeline and key investigations.

Year	Clinical event	Key investigations	Intervention
2014	ED visit: non-specific chest pain	Normal CBC	No hematologic treatment
Jan 2018	Abdominal pain, headache, back pain; splenic lesion identified	Hb 18 g/dL; JAK2 V617F and exon 12 negative; EPO 3.57 mIU/mL; splenic cystic lesion on US	Hydroxyurea 1 g/day + aspirin 100 mg/day initiated
2020–2022	Serial bone marrow biopsies; recurrent adherence lapses	BM: ~65–70% cellularity, trilineage proliferation, no dysplasia, no blasts; immunohistochemistry negative for malignancy; Hct ranging 38–57%	Repeated counseling; therapeutic phlebotomy during non-adherent phases
Jul 2022	Definitive molecular characterization; CT hepatomegaly	NGS myeloid panel: KMT2C c.2961C>G, p.Tyr987* VAF 21%; JAK2/CALR/MPL negative; CT liver craniocaudal 19.1 cm; splenic infarct calcifications	Hydroxyurea up-titrated to 2 g/day
Oct 2025	Stable hematological control; no further thrombotic events	Hb and Hct within target range; ongoing follow-up	Hydroxyurea 2 g/day continued; adherence reinforcement maintained

Hb, hemoglobin; EPO, erythropoietin; VAF, variant allele frequency; NGS, next-generation sequencing; ED, emergency department; BM, bone marrow; Hct, hematocrit.

## Diagnostic assessment, therapeutic intervention, follow-up, and outcomes

### Diagnostic assessment

#### Exclusion of secondary erythrocytosis

Secondary causes of erythrocytosis were systematically excluded before primary marrow disease was considered. Hypoxia-driven polycythemia (obstructive sleep apnoea, chronic pulmonary disease, high-altitude exposure) was not supported by clinical history or investigation. EPO-secreting tumors (renal cell carcinoma, hepatocellular carcinoma, cerebellar hemangioblastoma) were excluded by imaging and clinical context. Congenital erythrocytosis due to high-affinity hemoglobin variants or VHL/EPOR mutations was not supported by the clinical picture or family history. Serum erythropoietin was suppressed at 3.57 mIU/mL, supporting primary marrow-driven erythrocytosis.

#### Molecular workup

Repeated testing for JAK2 V617F and JAK2 exon 12 mutations was negative on multiple occasions. CALR and MPL mutations were also not detected. RNA-based fusion transcript analysis was not performed; however, the overall clinical and morphological constellation was not consistent with fusion-driven myeloid neoplasms. A myeloid NGS panel was subsequently applied and is described below. The full panel content included genes recurrently mutated in myeloid malignancies (including ASXL1, TET2, DNMT3A, EZH2, SF3B1, SRSF2, IDH1/2, TP53, and others); all were negative apart from the KMT2C variant reported here. No variants of uncertain significance were identified in any other gene on the panel; the KMT2C variant was the sole somatic alteration detected (**Table 2**).

**Table 2:** NGS result: identified pathogenic KMT2C variant.

Gene	cDNA Change	Protein change	VAF (%)	Consequence	Classification
KMT2C (MLL3)	c.2961C>G	p.Tyr987*	21%	Nonsense (premature stop codon)	Pathogenic (loss-of-function)

VAF, variant allele frequency; NGS, next-generation sequencing.

#### Bone marrow morphology

**Figure 2:**
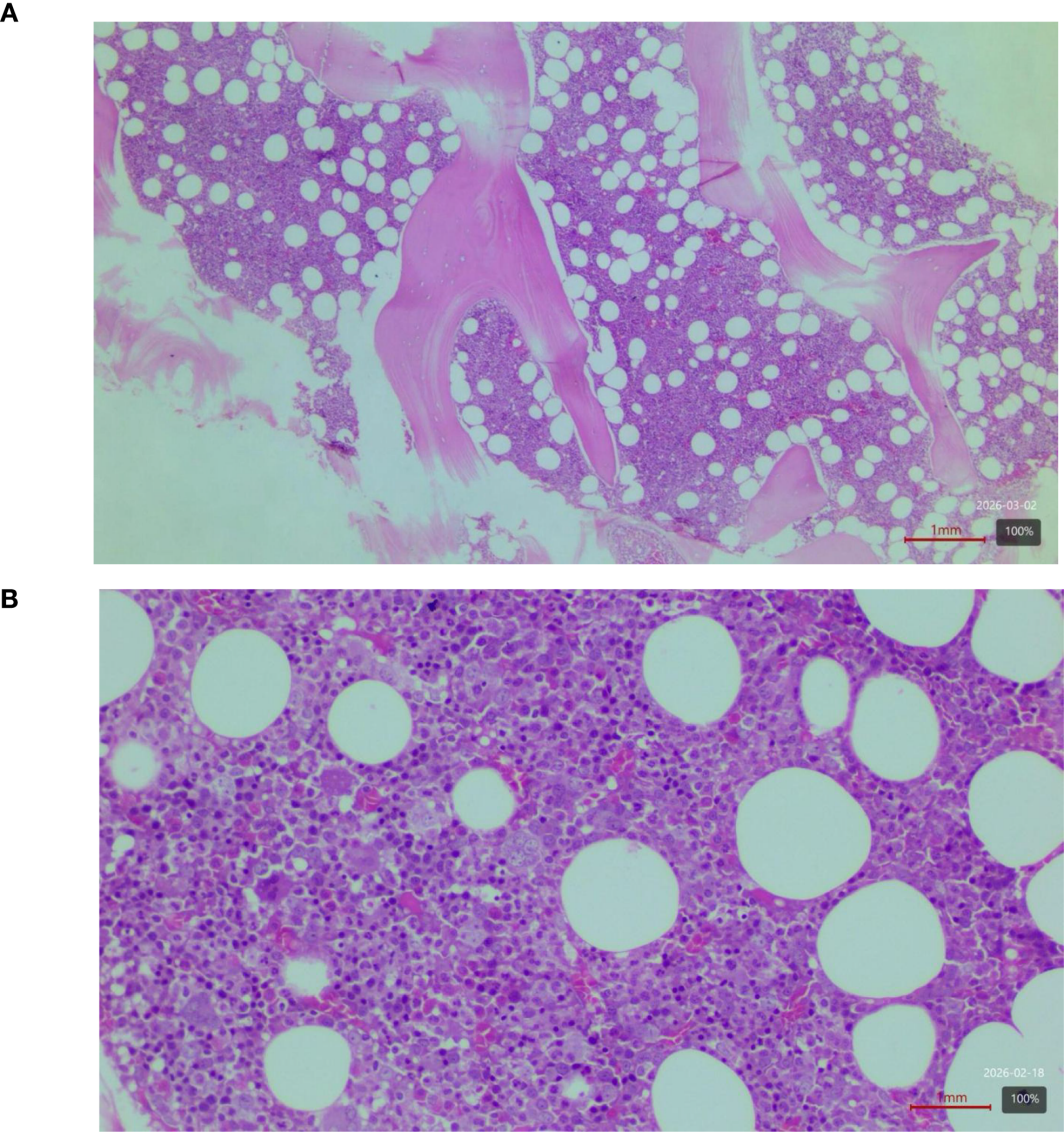
**(A)** Bone marrow core biopsy (hematoxylin and eosin stain; scale bar, 1 mm; original magnification, ×100). The biopsy shows mildly hypercellular marrow (~65–70% cellularity) relative to the patient’s age, with trilineage hematopoietic proliferation. The intertrabecular spaces demonstrate increased hematopoietic activity with reduced adipocyte volume. Bony trabeculae appear unremarkable without evidence of osteosclerosis or remodeling. **(B)** Higher-magnification view (hematoxylin and eosin stain; scale bar, 1 mm; original magnification, ×200) demonstrating increased erythroid precursors with normoblastic maturation. Megakaryocytes show no pleomorphism or abnormal clustering. No dysplasia, excess blasts, or reticulin fibrosis is identified. Histomorphological findings were reviewed by a consultant Hemopathologist and interpreted in the context of clinical, laboratory, and molecular data.

Serial bone marrow trephine biopsies (2020, 2021, 2022) were reviewed by a consultant hemopathologist. Sections demonstrated slight hypercellularity (~65–70% for the patient’s age) with trilineage proliferation, including increased erythroid precursors with normoblastic maturation and mild granulocytic expansion. Megakaryocytes show no significant pleomorphism or clustering. No significant dysplasia, increase in blasts, or reticulin fibrosis was identified. Immunohistochemical studies (CD3, CD20, c-Kit, CD34, TdT) showed no evidence of lymphoid or acute myeloid malignancy. While these marrow findings are supportive of an MPN-like process, we acknowledge that in a JAK2-negative setting, they are not pathognomonic for PV and must be interpreted in conjunction with the full clinical, laboratory, and molecular profile (**Figure 2**).

#### Differential diagnosis

The differential diagnosis in this case included: (i) JAK2-negative PV meeting clinical/morphological criteria; (ii) clonal erythrocytosis with an atypical molecular basis; (iii) MPN-unclassifiable; and (iv) atypical MPN not otherwise specified. Against clonal erythrocytosis or MPN-U: the sustained EPO suppression, panmyelotic marrow proliferation, documented thrombotic complications (splenic vein thrombosis), and hydroxyurea-responsiveness are collectively more consistent with a PV-like process than with a non-specific clonal erythrocytosis. We acknowledge that without a JAK2 mutation, the diagnostic label of PV cannot be applied with the same certainty as in mutation-positive cases. We therefore classify this case as a JAK2-negative PV-like MPN, in keeping with proposed frameworks for atypical erythrocytosis (1–3, 7).

#### Molecular findings

Definitive molecular characterization was achieved in July 2022, when a myeloid NGS panel identified a nonsense mutation in the KMT2C gene. The KMT2C nonsense variant (p.Tyr987*, rs58528565) is predicted to result in protein truncation. It is absent from large population databases such as gnomAD, supporting its rarity (8). This variant has been reported in ClinVar, where it is currently classified as a variant of uncertain significance, highlighting the need for cautious interpretation despite its predicted loss-of-function effect. In contrast, *KMT2C* alterations are frequently reported in cancer databases such as COSMIC, supporting the potential role of loss-of-function variants in oncogenesis (5, 6, 9). Integrating the clinical presentation, suppressed serum EPO, morphological findings, and molecular evidence of clonality, this case is best described as a JAK2-negative PV-like MPN within the conceptual framework of the 2022 WHO classification (1, 2).

### Therapeutic intervention

Hydroxyurea was initiated at 1 g daily for cytoreduction, targeting a hematocrit below 45% per current PV management guidelines (10). Low-dose aspirin (100 mg daily) was added for thromboprophylaxis. Persistent heavy smoking was addressed through repeated counseling for smoking cessation. Due to incomplete hematological control during non-adherent phases, the hydroxyurea dose was up-titrated to 2 g daily.

### Follow-up and outcomes

Following dose adjustment and improved adherence, stable hematological control was achieved. As of October 2025, the patient remains on hydroxyurea 2 g daily with stable blood counts and no further thrombotic events. Ongoing follow-up is maintained for disease monitoring and adherence reinforcement.

## Discussion

### Diagnostic classification and the challenge of JAK2-negative erythrocytosis

JAK2 mutations are central to the 2022 WHO and 2023 ICC diagnostic criteria for PV, and their absence fundamentally changes the diagnostic calculus (1–3). In this patient, repeated negativity for JAK2 V617F and exon 12 mutations, combined with absent CALR and MPL alterations, placed the case within a diagnostically challenging category (11). Rather than asserting a definitive PV diagnosis, we classify this case as a *JAK2-negative PV-like MPN*, reflecting the morphological and clinical convergence with PV in the absence of the molecular pillar that anchors that diagnosis.

The differential diagnosis in JAK2-negative erythrocytosis is broad and must be explicitly addressed. Secondary erythrocytosis was rigorously excluded through clinical assessment and imaging. Fusion-driven myeloid neoplasms were considered; while formal RNA-based fusion testing was not performed, the clinical and morphological constellation was not consistent with such entities, and this represents an acknowledged limitation that limits the ability to fully exclude rare fusion-driven myeloid neoplasms. Among primary clonal processes, MPN-unclassifiable and clonal erythrocytosis of uncertain significance were carefully considered but deemed less consistent with the overall phenotype, given the sustained EPO suppression, panmyelotic marrow expansion, thrombotic complications, and cytoreductive treatment responsiveness. We recognize, however, that this classification carries inherent uncertainty in the absence of a canonical molecular driver, and the case could reasonably be categorized as atypical MPN pending further molecular evidence.

### Molecular insights: KMT2C as a clonal marker and candidate contributor

The KMT2C p.Tyr987* variant introduces a premature stop codon, resulting in truncation of the MLL3 protein and likely loss of histone H3K4 monomethyltransferase activity, a recognized mechanism of KMT2C inactivation (5, 6). This variant was absent from population databases and catalogued in COSMIC in hematologic malignancy contexts, supporting its somatic and potentially oncogenic nature. KMT2C mutations have been reported in clonal hematopoiesis of indeterminate potential (CHIP) and in acute myeloid leukemia (5, 6), but their association with a PV-like erythrocytosis phenotype has not previously been documented.

Importantly, we emphasize that this case establishes a correlative association, not a causal relationship. A single case report cannot determine whether KMT2C p.Tyr987* is a founding driver mutation sufficient to cause a PV-like phenotype, a cooperating lesion acting alongside an undetected primary event, or an incidental clonal event that is not mechanistically linked to the erythroid expansion. This distinction is critical and cannot be resolved without functional validation. Future studies addressing this question could include: (i) CRISPR-mediated KMT2C knockout in hematopoietic progenitor cell lines to assess erythroid differentiation bias; (ii) generation of murine models carrying analogous KMT2C truncating mutations; or (iii) directed differentiation of patient-derived induced pluripotent stem cells (iPSCs) to hematopoietic lineages to characterize clonal behavior *in vitro*.

Disruption of KMT2C (MLL3)-mediated enhancer regulation and H3K4 monomethylation has been associated with altered transcriptional control in hematopoietic stem cells and may contribute to clonal expansion of erythroid-biased progenitors. In this context, the identified variant could represent a candidate epigenetic contributor to a PV-like phenotype. Impaired enhancer regulation may lead to activation of pathways that phenotypically mimic JAK–STAT signaling, such as through derepression of cytokine-regulated genes. However, this remains an open and experimentally testable question that cannot be resolved from clinical data alone. Accordingly, any biological or therapeutic implications are speculative and should not be extrapolated to clinical practice in the absence of functional validation and independent replication.

### Clinical management and adherence

This case underscores the substantial impact of treatment non-adherence on PV outcomes. Hematocrit oscillations between 38% and 57% translated into wide fluctuations in thrombotic risk, consistent with data from the CYTO-PV trial, which demonstrated that maintaining hematocrit below 45% significantly reduced the rate of cardiovascular death and major thrombosis (12). The case reinforces current European LeukemiaNet and NCCN guidelines designating hematocrit maintenance below 45% as the primary cytoreductive target in PV (10). Dose adjustment of hydroxyurea from 1 g to 2 g daily achieved sustained hematological control, highlighting that dose optimisation is the appropriate response to breakthrough erythrocytosis during periods of adherence.

### Strengths and limitations

Strengths include rigorous and systematic exclusion of secondary erythrocytosis, longitudinal documentation over seven years, review of bone marrow morphology by a consultant hematopathologist, and use of a myeloid NGS panel to establish molecular evidence of clonality with identification of a somatic, absent-from-population-databases variant in a known myeloid tumor suppressor gene.

Limitations include: the inherent inability to establish causal inference from a single case report; the absence of RNA-based fusion transcript analysis;the absence of serial NGS to monitor clonal evolution and longitudinal VAF dynamics, which would have been valuable to distinguish a stable Clonal hematopoiesis of indeterminate potential-associated event from a progressive disease-driving clone; the use of a targeted myeloid panel that may not have captured all co-occurring variants; and the lack of functional validation of the KMT2C variant in hematopoietic models. These limitations are acknowledged explicitly and define the scope of conclusions that can responsibly be drawn.

### Take-away lessons

First, JAK2-negative erythrocytosis is a diagnostically heterogeneous category, and comprehensive genomic profiling is essential to establish clonality and guide differential diagnosis. Second, KMT2C loss-of-function represents a candidate molecular marker in atypical JAK2-negative erythrocytosis, though causal contribution remains to be demonstrated. Third, the association between KMT2C mutation and a PV-like phenotype is correlative in this single case and requires replication and functional validation before mechanistic conclusions are drawn. Fourth, strict adherence to cytoreductive therapy and guideline-based hematocrit targets is critical to prevent thrombotic complications, and structured patient education with multidisciplinary support should be prioritized in patients at risk of non-adherence.

### Patient perspective

The patient reported significant relief following the establishment of a definitive diagnosis after several years of unexplained symptoms. He acknowledged periods of difficulty maintaining strict adherence to his medication regimen, often attributing this to feeling well during controlled phases of his illness. He expressed understanding of the relationship between hematocrit levels and thrombotic risk following sustained patient education, and committed to ongoing follow-up and medication adherence. Written informed consent for publication was provided.

## Conclusion

This case identifies KMT2C p.Tyr987* as a somatic clonal marker in a patient with a JAK2-negative PV-like MPN phenotype characterized by sustained erythrocytosis, suppressed EPO, panmyelotic bone marrow morphology, and thrombotic complications. The observed association between KMT2C loss-of-function and this clinical phenotype is correlative; causality cannot be established from a single case and functional validation is required. This observation expands the known molecular landscape of JAK2-negative MPNs and highlights the pivotal role of comprehensive genomic profiling when canonical driver mutations are absent. Future case registries and collaborative genomic databases of JAK2-negative MPNs will be essential to determine whether KMT2C loss-of-function represents a recurrent molecular finding in this diagnostic category.

## Data Availability

The original contributions presented in the study are included in the article/supplementary material. Further inquiries can be directed to the corresponding author.
